# Study on the Effects and Mechanisms of Fly Ash, Silica Fume, and Metakaolin on the Properties of Slag–Yellow River Sediment-Based Geopolymers

**DOI:** 10.3390/ma18081845

**Published:** 2025-04-17

**Authors:** Ge Zhang, Kunpeng Li, Huawei Shi, Chen Chen, Chengfang Yuan

**Affiliations:** 1 Yellow River Institute of Hydraulic Research, Yellow River Water Conservancy Commission, Zhengzhou 450003, China; gezhangyrihr@163.com (G.Z.); 15538352232@163.com (H.S.); 15617633649@163.com (C.C.); 2 Key Laboratory of Lower Yellow River Channel and Estuary Regulation, Ministry of Water Resources, Zhengzhou 450003, China; 3 Yellow River Laboratory, Zhengzhou 450003, China; 4 College of Civil Engineering, Zhengzhou University, Zhengzhou 450001, China

**Keywords:** Yellow River sediment, geopolymers, strength, characteristic products, microstructure

## Abstract

The incorporation of mineral admixtures plays a crucial role in enhancing the performance and sustainability of geopolymer systems. This study evaluates the influence of fly ash (FA), silica fume (SF), and metakaolin (MK) as typical mineral admixtures on slag–Yellow River sediment geopolymer eco-cementitious materials. The impact of varying replacement ratios of these admixtures for slag on setting time, workability, reaction kinetics, and strength development were thoroughly investigated. To understand the underlying mechanisms, microstructural analysis was conducted using thermogravimetric–differential thermal analysis (TG-DTA), X-ray diffraction (XRD), scanning electron microscopy–energy dispersive spectroscopy (SEM-EDS), and mercury intrusion porosimetry (MIP). The results indicate that the incorporation of FA, SF, and metakaolin delayed the initial reaction, prolonged the induction period, and reduced the acceleration rate. These effects hindered early strength development. At 30% FA content, the matrix exhibited excellent flowability and sustained heat release. The 28-day splitting tensile strength increased by 42.40%, while compressive strength decreased by 2.85%. In contrast, 20% SF significantly improved compressive strength, increasing the 28-day compressive and splitting tensile strengths by 11.19% and 6.16%, respectively. At 15% metakaolin, the strength improvement was intermediate, with 28-day compressive and splitting tensile strengths increasing by 3.55% and 10.59%, respectively. However, dosages exceeding 20% for SF and metakaolin significantly reduced workability. The incorporation of FA, SF, and metakaolin did not interfere with the slag’s alkali-activation reaction. The newly formed N-A-S-H and C-S-H gels integrated with the original C-A-S-H gels, optimizing the pore structure and reducing pores larger than 1 µm, enhancing the matrix compactness and microstructural reinforcement. This study provides practical guidance for optimizing the use of sustainable mineral admixtures in geopolymer systems.

## 1. Introduction

The protection and sustainable management of the Yellow River is a critical priority for the long-term development of China [1]. The Yellow River Basin is facing severe sedimentation issues, exacerbated by factors such as low water flow, excessive sediment load, and the imbalance between water and sediment transport [2,3]. Sediment deposition has long been a pressing technical challenge, particularly in flood control, maintaining the effective storage capacity of reservoirs, and ensuring the stable operation of irrigation systems. Meanwhile, the demand for construction aggregates in China is substantial, with approximately 20 billion tons of sand and gravel consumed annually [4,5]. As natural sand resources are increasingly depleted, the search for viable alternatives has become imperative. In this context, YRS, predominantly composed of silicon dioxide (SiO_2_), emerges as a promising substitute for conventional construction materials. Leveraging these sediment resources not only presents significant socio-economic benefits but also contributes to ecological sustainability.

**Figure 1:**
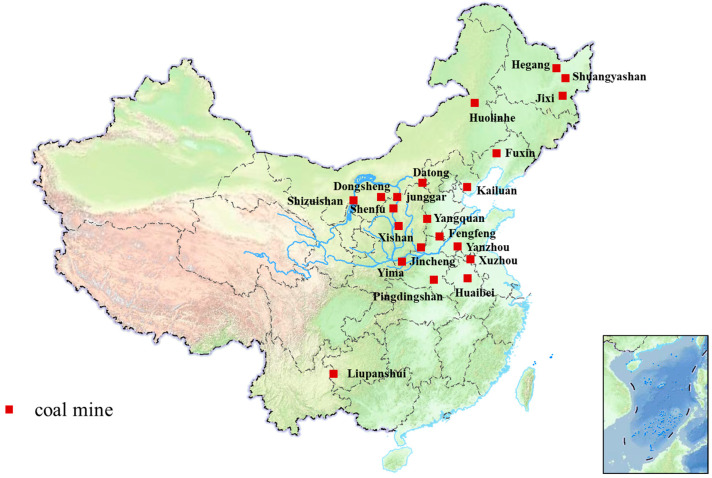
Distribution map of major coal mines in China.

The Yellow River Basin is renowned as China’s “Energy Basin” and serves as a vital energy and industrial base for the country, supporting a significant portion of the country’s energy and manufacturing sectors [6]. Nine of the country’s fourteen major coal production bases are located within this basin, as shown in Figure 1, while heavy industries such as steel and aluminum production dominate the provinces of Shanxi, Henan, and Shandong [7,8]. These industrial production bases have made significant contributions to the economic development of the regions along the Yellow River, driving regional industrialization and urbanization. However, large-scale industrial activities have also brought about notable negative impacts, particularly the generation of substantial amounts of solid waste, such as fly ash and slag. The long-term accumulation of these solid wastes not only occupies vast land resources but also causes severe pollution of soil, water bodies, and the atmosphere, damaging the local ecological environment [9].

Geopolymer (also known as alkali-excited cementitious material) is characterized by fast setting speed [10,11], high-temperature resistance [12,13,14], and corrosion resistance [15,16,17]. Geopolymer can be synthesized without the need for calcination, offering significant potential for reducing carbon emissions by replacing conventional cement [18,19,20,21]. As a crucial component in geopolymer systems, mineral admixtures play a pivotal role in modifying the properties of alkali-activated cementitious materials [22,23,24,25], which are mainly reflected in the reaction process and characteristic products of the matrix, and the pore structure and microstructure are closely related to macroscopic properties. Slag–Yellow River sediment geopolymer is a novel green building material with excellent mechanical properties, durability, and environmental friendliness. However, its performance is significantly influenced by the raw material characteristics, mix proportions, and reaction conditions. To further enhance its performance, the incorporation of mineral admixtures such as fly ash [26,27,28,29], metakaolin [30,31,32,33], and silica fume [34,35,36,37,38] has become an effective optimization strategy.

Wang et al. used Yellow River silt as a raw material, combined with blast furnace slag, to prepare a cementitious material with a strength of up to 12.3 MPa [39]. He et al. studied the combination of Yellow River silt and red mud for the preparation of sintered bricks with a strength of up to 39 MPa and studied the characteristics and mechanism of the preparation process [40]. Sheng Jiang incorporated FA and utilized alkali activation to fabricate artificial stone. The study revealed that the alkali activator, FA dosage, and curing age significantly contributed to the enhancement of mechanical properties. The highest compressive strength of the artificial stones at 90 days was 16.6 MPa, with the corresponding splitting tensile strength being 1.4 MPa [41]. Feng Hu et al. utilized fly ash in the preparation of engineered geopolymer composites (EGCs), and the resulting material has been effectively implemented in the construction of subway tunnel shield segments [42]. Sheng Jiang utilized YRS and fly ash to prepare alkali-activated fly ash (AAFA) foam concrete [43]. These findings demonstrate the feasibility of using Yellow River sand as a reliable and cost-effective building material in the production of alkali-activated fly ash (AAFA) foam concrete. Weizhun Ji utilized red mud and YRS to prepare alkali-activated cementitious materials and investigated their mechanical properties [44]. The research findings revealed that the combination of RM and YRS promotes the chemical reaction of the Al and Si elements and generates the (N, C)-A-S-H gel products, which are the key to the strength enhancement of the cementitious material.

Yellow River sediment exhibits notable differences from conventional construction sand in terms of particle size distribution and physicochemical characteristics. Its particles are predominantly fine, mainly within the silt and clay particle range (<0.075 mm), with flaky or angular shapes, rough surfaces, large specific surface areas, and high clay mineral contents. These characteristics result in distinct performance differences when used as aggregate in geopolymers compared with those using traditional river sand. Therefore, existing research findings cannot be directly applied to predict the performance of alkali-activated Yellow River sediment eco-cementitious materials. It is necessary to establish a dedicated parameter optimization system specifically tailored to the unique properties of Yellow River sediment. Currently, research on the influence of mineral admixtures on the performance of slag–YRS geopolymers remains limited and lacks a systematic approach, particularly regarding the synergistic effects of multiple mineral admixtures. This gap in understanding hinders the ability to provide comprehensive guidance for engineering applications. Based on the aforementioned background, this study utilized FA, SF, and MK as typical mineral admixtures. By setting different replacement ratios to substitute slag, the influence of the types and replacement ratios of mineral admixtures on the setting time, workability, reaction process, and strength of slag–Yellow River sediment geopolymer eco-friendly cementitious materials were systematically investigated. Furthermore, combined with microstructural testing techniques such as TG-DTA, XRD, SEM-EDS, and MIP, the influence mechanisms of the mineral admixtures on the characteristic products, pore structure, and microstructure of the matrix were revealed. Through this research, the study aims to provide theoretical foundations and technical references for the research and application of multi-source solid waste–Yellow River sediment geopolymers.

## 2. Materials and Methods

### 2.1. Raw Material and Mixed Proportion

#### 2.1.1. Raw Material

**Figure 2:**
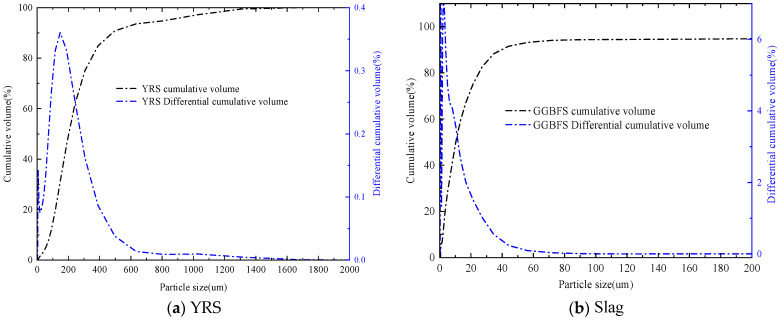
Particle size distribution curves of YRS and slag.

**Figure 3:**
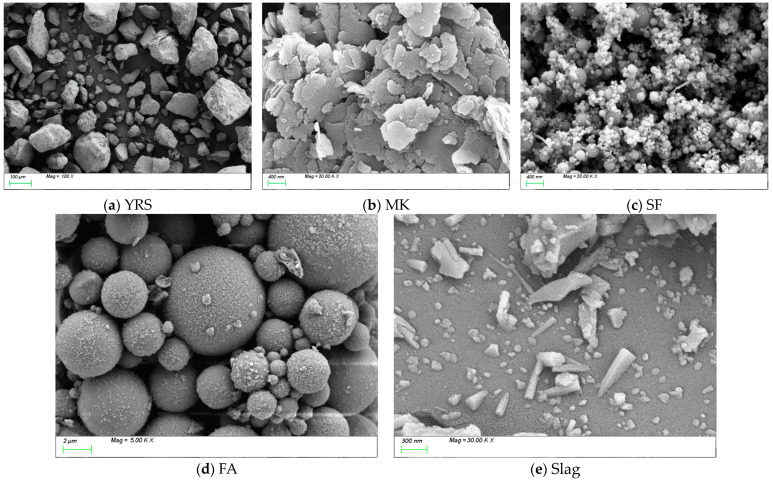
SEM images of YRS and mineral admixtures.

The raw materials used in this study primarily included YRS, FA, slag, SF, MK, sodium hydroxide (NaOH), and water glass. The YRS was sourced from the Xixiayuan Reservoir in Henan Province, initially in a moist state, and it was then dried before use. The median particle size of the YRS was determined to be 0.20 mm. Grade I fly ash, with a density of 2.18 g/cm^3^, was utilized in the experiments. The slag exhibited a median particle size of 10.28 μm, a hydraulic coefficient of 2.17, an activity coefficient of 0.48, and an alkalinity coefficient of 1.14. SF had a specific surface area ranging from 18,000 to 20,000 m^2^/kg, a density of 2.214 g/cm^3^, a SiO_2_ content of ≥92%, and an average particle size of 15.78 μm. Highly reactive metakaolin was used, with 7-day and 28-day activity indices of 115% and 118%, respectively, and a median particle size of 4.98 μm. The chemical compositions of the YRS, FA, MK, slag, and SF were analyzed using X-ray fluorescence (XRF), and the results are provided in Table 1. The particle sizes of the YRS and slag were analyzed using Malvern Mastersizer-2000 laser particle-size analyzer (Malvern Panalytical, Worcestershire, UK). The results are presented in Figure 2. SEM images, shown in Figure 3, revealed that YRS particles displayed irregular geometric shapes, with considerable variation in particle size. The mineral admixtures exhibited diverse morphologies: MK was primarily observed as flaky accumulations, while SF and FA formed spherical aggregates. In contrast, slag particles demonstrated a heterogeneous morphology, consisting of irregular shapes, flakes, spheres, and angular fragments. Water glass, also known as sodium silicate (Na_2_O·nSiO_2_), is a transparent, glassy solution made up of alkali metal silicates. The chemical composition of water glass is defined by the molecular ratio n between SiO_2_ and alkali metal oxides, Na_2_O or K_2_O, referred to as the water glass modulus. In this study, liquid sodium silicate produced by Shandong Yourui Chemical Co., Ltd. (Weifang, China) was used, with its physicochemical parameters outlined in Table 2. The water glass modulus was adjusted to 1.2 using pure NaOH, as indicated by the modulus adjustment equation in Formula (1). Tap water was employed as the mixing water throughout the experiments.

(1) $${\mathrm{Na}}_{2}O\cdot 2.18SiO_{2}+NaOH\to Na_{2}O\cdot nSiO_{2}+H_{2}O$$

#### 2.1.2. Mixed Proportion

Before the test, the pre-measured NaOH was thoroughly blended into the sodium silicate. The mixture was prepared using a single horizontal shaft concrete mixer. Initially, the YRS and mineral admixtures were added to the mixer and stirred for 180 s. Water was then added, and the mixture was stirred for another 180 s. Following this, water glass was incorporated, and the mixture was stirred for 120 s. The mixture was then quickly cast into molds and consolidated on a vibration table for 60 to 90 s.

The setting time and workability are the key indices to measure whether a mixture is easy to transport, pour, vibrate, and form, which plays an extremely important role in ensuring the construction quality. This study first analyzed the setting time, workability, and early-stage reaction characteristics to determine the optimal dosage ranges of mineral admixtures (FA, SF, MK) while ensuring satisfactory workability. Subsequently, a systematic investigation was conducted on the effects of these admixtures on mechanical strength coupled with mechanistic analysis. The setting time and workability proportions are presented in Table 3, the hydration heat proportions are presented in Table 4, and the strength and microcosmic proportions are presented in Table 5. All mix proportions in this study were formulated on mass ratio.

### 2.2. Experimental Method

In this experiment, the effects of FA, SF, and MK dosage on the workability, mechanical properties, reaction progress, characteristic products, and microstructural properties of slag–Yellow River sediment-based geopolymers were analyzed. The analysis included setting time, compressive and splitting tensile strength, hydration heat, thermogravimetric analysis, XRD, MIP, and SEM. Table 6 presents the test grouping, including the size and number of each specimen and test.

#### 2.2.1. Setting Time Test

Due to the fact that a high content of alkali can significantly accelerate the setting of the paste, conventional methods for measuring the setting time of cement are not suitable for alkali-activated pastes. The setting time was tested according to GB/T 35159 (flash setting admixtures for shotcrete) [45]. The water–mineral admixture ratio used for determining the setting time of the paste was 0.35, and the average value was taken from three experiments in each group.

#### 2.2.2. Workability Test

The workability of the mixture was tested using a micro-collapse cylinder with an upper mouth of 50 mm, a lower mouth of 100 mm, a height of 150 mm, and a 60 cm × 60 cm plate.

#### 2.2.3. Hydration Heat Test

The hydration heat was measured using a TAM AIR isothermal calorimeter (TA Instruments, New Castle, DE, USA). The test was conducted at a temperature of 25 °C, with a temperature fluctuation range of less than 0.02 °C and a measurement accuracy of ±20 μW. The duration of the test was 4 days.

#### 2.2.4. Strength Test

In order to fully evaluate the effect of fiber on the strength and toughness of the matrix, cube specimens with side lengths of 100 mm were used to test their compressive strength and splitting tensile strength. The specimens were then placed in a standard curing room (20 °C ± 2 °C and RH > 95%) to be cured until they reached the specified age for testing, following the requirements outlined in GB/T 50081-2019, “Standard for Test Methods of Physical and Mechanical Properties of Concrete” [46]. The tension–compression ratio was calculated according to the test results.

#### 2.2.5. X-Ray Diffraction Analysis

Using the Japanese physical X-ray diffractometer, the sample was taken after drying and grinding treatment and put into the glass groove for testing. The sampling interval was 0.04° (2θ), the sampling speed was 2°/min, and the scanning angle range was 5°–70° (2θ).

#### 2.2.6. Thermogravimetric Analysis

A ZCT-B simultaneous thermal analyzer (Beijing Jingyi Hitechinstrument Co., Ltd., Beijing, China) was employed, with the test sample weighing approximately 15 mg. The heating rate was controlled at 10 °C/min, and the maximum temperature was raised to 1000 °C. The DTA curves for each group of samples were obtained.

#### 2.2.7. Porosity Test

The porosity and pore size distribution of the sample were analyzed using the mercury intrusion method. Small paste specimens were prepared for standard curing (20 °C ± 2 °C, RH > 95%). After curing for 28 days, the hydrated samples were broken using pliers for testing. To ensure the reliability of test results, all specimens were randomly sampled, with each group measured in triplicate, and the arithmetic mean of the three measurements was adopted as the final value. The porosity was measured using a Poremaster-33T automatic mercury porosimeter (Anton Paar Quanta Tec Inc., Boynton Beach, FL, USA) with an aperture measurement range of 3.5 nm to 360,000 nm.

#### 2.2.8. Scanning Electron Microscopy Test

The microstructure of the samples was observed using a Sigma 300 field emission environmental scanning electron microscope (Carl Zeiss AG, Oberkochen, Germany). After curing to the 28 d age, the samples were broken with pliers, and hydration was stopped before testing [47].

## 3. Experiment Results and Analysis

### 3.1. Setting Time

**Figure 4:**
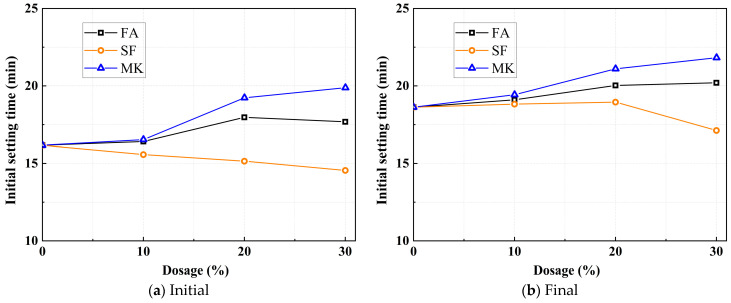
Effect of mineral admixture on setting time.

Figure 4 illustrates the effect of varying mineral admixtures on the setting time of the paste. Due to the high alkali content in the mixture slurry, both the initial and final setting times for all mix proportions do not exceed 25 min, indicating a relatively rapid reaction rate. Figure 4a specifically shows the influence of different mineral admixtures on the initial setting time of the paste.

As shown in Figure 4, different mineral admixtures exert distinct effects on the initial setting time. The initial setting time increases with higher dosages of FA and MK, while it decreases with higher SF content. For example, at a 30% FA and MK dosage, the initial setting time increases from 16.17 min to 17.18 min and 19.88 min, respectively. Conversely, when the SF dosage reaches 30%, the initial setting time decreases from 16.17 min to 14.55 min. This is attributed to the relatively low pozzolanic activity of FA, which results in slower early-stage reactions, while its particle-induced “ball-bearing effect” enhances the fluidity of the slurry, thereby delaying the setting process. MK, exhibiting lower pozzolanic activity than slag, also retards the setting process. SF, characterized by its high specific surface area and pronounced pozzolanic reactivity, rapidly reacts with alkaline activators to form C-S-H, thereby accelerating early hydration reactions and expediting the setting process.

Figure 4b illustrates the influence of mineral admixture dosage on the final setting time of the paste. As shown in the figure, similar to the pattern observed in the initial setting time, the final setting time increases with higher dosages of FA and MK, while it decreases with elevated SF content. At 30% FA and MK, the final setting time increases from 18.62 min to 20.20 min and 21.82 min, respectively. However, when the SF content reaches 30%, the final setting time decreases from 18.62 min to 14.55 min. This behavior is attributed to the slower reaction kinetics of FA, coupled with its ability to enhance the fluidity of the mixture. The lamellar stacking structure of MK, with its high specific surface area, entraps free water between the particles. As the mixture’s workability decreases, this stored free water is released, delaying the solidification of the slurry. SF, on the other hand, significantly shortens the final setting time by accelerating the overall reaction kinetics due to its elevated pozzolanic reactivity.

### 3.2. Workability

**Figure 5:**
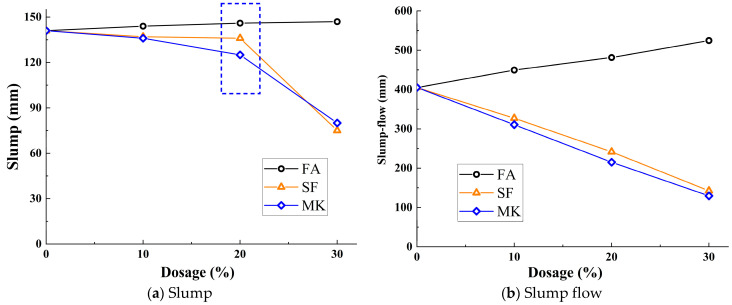
Effect of mineral admixture on workability.

Figure 5 illustrates the effect of varying replacement ratios of mineral admixtures on the slump and slump flow of the mixture. Specifically, Figure 5a shows the influence of different mineral admixture replacement ratios on the slump. As observed in Figure 5a, distinct patterns emerge in the influence of different mineral admixtures on the slump. The slump increases with higher dosages of FA. At a 30% FA dosage, the slump rises from 141 mm to 147 mm, though the increase is not statistically significant. This behavior is primarily attributed to the inherent fluidity of the alkali-activated slag system, which results in minimal slump loss. Conversely, as shown in the figure, the slump decreases with increasing dosages of SF and MK. Notably, when the SF and MK dosage increases from 20% to 30%, the slump significantly decreases, from 136 mm and 125 mm to 75 mm and 80 mm, respectively.

Figure 5b illustrates the influence of different mineral admixture replacement ratios on the slump flow of the mixture. Similar to the influence pattern observed in a slump, the slump flow of the mixture increases with higher dosages of FA. When the FA dosage reaches 30%, the slump flow increases from 405 mm to 524.5 mm. As also indicated by the figure, contrary to the trend observed with FA, the slump flow of the mixture significantly decreases with the increase in SF and MK dosage. When the dosage is increased to 30%, the slump flow of the mixture decreases from 405 mm to 142.5 mm and 129.5 mm, respectively.

In summary, both the slump and slump flow of the mixture increase with higher FA dosages and decrease with higher dosages of SF and MK. When the dosages of SF and MK exceed 20%, the reduction in slump becomes significantly more pronounced. This is attributed to the spherical shape of the FA particles, which exhibit a “ball-bearing effect” that enhances the fluidity of the paste. Furthermore, the filling effect of FA reduces interparticle friction, further improving the slump. In contrast, MK, with its high water absorption capacity, adsorbs substantial amounts of moisture, resulting in reduced paste fluidity. Additionally, the platelet morphology of MK intensifies interparticle friction, leading to a further reduction in slump. Similarly, SF, with its high specific surface area, absorbs significant water content, thus reducing paste fluidity. The micro-filling effect of SF also increases interparticle contact, further decreasing slump. Therefore, to maintain acceptable workability, the dosage of SF and MK should not exceed 20%.

### 3.3. Reaction Process

**Figure 6:**
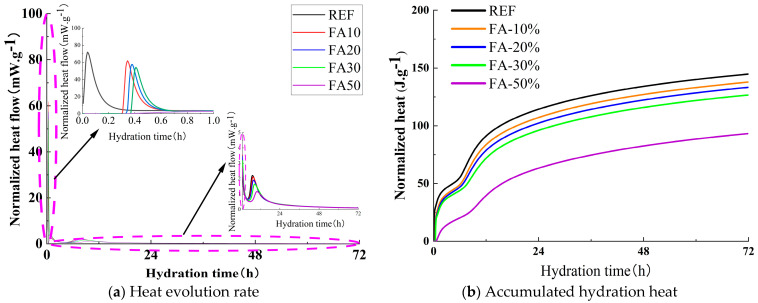
Effect of FA on heat release curve.

**Figure 7:**
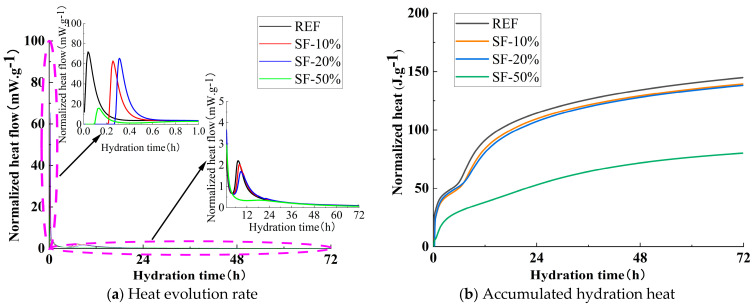
Effect of SF on heat release curve.

**Figure 8:**
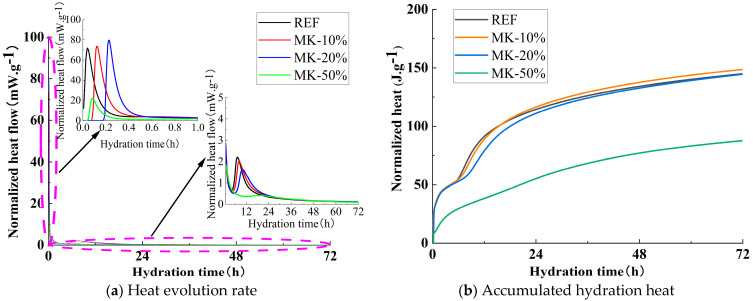
Effect of MK on heat release curve.

Distinct from conventional cementitious materials, geopolymers exhibit more intense early-stage reactions. Isothermal calorimetry can systematically analyze the reaction characteristics upon incorporation of mineral admixtures (e.g., FA, SF, MK), thereby elucidating their effects on the “depolymerization–condensation” process in geopolymer systems. Figure 6, Figure 7 and Figure 8 illustrate the influence of FA, SF, and MK dosages on the early reaction kinetics of the mixture, respectively. Figure 6 specifically demonstrates the effect of FA content on the hydration heat release rate and cumulative hydration heat. The hydration exothermic process of alkali-activated binders can be divided into five stages: initial dissolution period, induction period, acceleration period, deceleration period, and stabilization period. The hydration exothermic curve of alkali-activated slag cement exhibits a primary initial peak and a secondary initial peak before the induction period followed by an acceleration peak after the induction period. This phenomenon occurs because the primary and secondary initial peaks emerge in close temporal proximity, eventually merging into a single peak. The primary initial peak is attributed to the wetting and dissolution of slag particles, while the secondary peak arises from reactions between Ca^2^⁺ ions dissolved from slag and anionic species released from the sodium silicate solution. These reactions and their resulting products, primarily C-A-S-H gel, play a critical role in governing the setting time and strength development of the matrix. The substantial precipitation of C-A-S-H gel marks the onset of the induction period, while the acceleration peak corresponds to the increased reaction kinetics of slag particles [48].

Figure 6a illustrates the influence of FA dosage on the hydration heat release rate. As shown in the figure, unlike the reference group, which initiated the reaction immediately, FA10, FA20, and FA30 began their initial reactions at 0.30 h, 0.34 h, and 0.37 h, respectively. The normalized heat flow directly reflects the reaction intensity. The normalized heat flow values for the initial reaction peaks of the reference group, FA10, FA20, FA30, and FA50 were 71.54 mW/g, 61.60 mW/g, 57.34 mW/g, 53.95 mW/g, and 2.69 mW/g, respectively. Compared with the reference group, the peak intensities of FA10, FA20, FA30, and FA50 decreased by 13.89%, 19.85%, 24.59%, and 96.24%, respectively. During the acceleration period, the normalized heat flow values for the acceleration peaks of the reference group, FA10, FA20, FA30, and FA50 were 2.21 mW/g, 2.11 mW/g, 1.91 mW/g, 1.65 mW/g, and 1.16 mW/g, respectively. The peak intensities of FA10, FA20, FA30, and FA50 decreased by 4.52%, 13.57%, 25.34%, and 47.51%, respectively, compared with the reference group. In summary, the incorporation of FA delayed the onset of the initial reaction period, extended the duration of the induction period, and reduced the reaction rates during both the initial reaction and acceleration periods. Additionally, the reaction rate decreased as the FA dosage increased.

Figure 6b illustrates the influence of FA dosage on the cumulative hydration heat. As shown in the figure, the cumulative hydration heat decreases with increasing FA dosage. After 24 h of cumulative heat release, the reference group exhibited a hydration heat of 114.4 J/g, while FA10, FA20, FA30, and FA50 showed hydration heats of 107.2 J/g, 102.4 J/g, 96.1 J/g, and 63.4 J/g, respectively. These values represent reductions of 6.27%, 10.50%, 15.98%, and 44.60% compared with the reference group. After 72 h of cumulative heat release, the reference group exhibited a hydration heat of 144.8 J/g, while FA10, FA20, FA30, and FA50 showed hydration heats of 137.8 J/g, 133.3 J/g, 126.7 J/g, and 93.2 J/g, respectively. These values represent reductions of 4.79%, 7.93%, 12.50%, and 35.59% compared with the reference group.

Figure 7a illustrates the influence of SF dosage on the hydration heat release rate. As shown in the figure, unlike the reference group, which initiated the reaction immediately, SF10, SF20, and SF50 began their initial reactions at 0.26 h, 0.31 h, and 0.14 h, respectively. The normalized heat flow values for the initial reaction peaks of the reference group, SF10, SF20, and SF50 were 71.54 mW/g, 62.46 mW/g, 65.41 mW/g, and 16.00 mW/g, respectively. Compared with the reference group, the peak intensities of SF10, SF20, and SF50 decreased by 12.69%, 8.57%, and 77.63%, respectively. During the acceleration period, the normalized heat flow values for the acceleration peaks of the reference group, SF10, and SF20 were 2.21 mW/g, 2.04 mW/g, and 1.70 mW/g, respectively, while SF50 did not exhibit a discernible acceleration peak. The peak intensities of SF10 and SF20 decreased by 7.69% and 23.08%, respectively, compared with the reference group. Overall, similar to the trend observed with FA, the incorporation of SF delayed the onset of the initial reaction period, prolonged the induction period, and reduced the reaction rates in both the initial reaction and acceleration periods.

Figure 7b illustrates the influence of SF dosage on the cumulative hydration heat. As shown in the figure, after 24 h of cumulative heat release, the reference group exhibited a hydration heat of 114.4 J/g, while SF10, SF20, and SF50 showed hydration heats of 109.5 J/g, 107.3 J/g, and 52.9 J/g, respectively. These values represent reductions of 4.25%, 6.23%, and 53.71% compared with the reference group. After 72 h of cumulative heat release, the reference group exhibited a hydration heat of 144.8 J/g, while SF10, SF20, and SF50 showed hydration heats of 139.3 J/g, 138.1 J/g, and 80.1 J/g, respectively. These values represent reductions of 3.77%, 4.58%, and 52.9% compared with the reference group.

Figure 8a illustrates the influence of MK dosage on the hydration heat release rate. As shown in the figure, unlike the reference group, which initiated the reaction immediately, MK10, MK20, and MK50 began their initial reactions at 0.13 h, 0.23 h, and 0.09 h, respectively. The normalized heat flow values for the initial reaction peaks of the reference group, MK10, MK20, and MK50 were 71.54 mW/g, 73.61 mW/g, 79.69 mW/g, and 22.06 mW/g, respectively. Compared with the reference group, the peak intensities of MK10 and MK20 increased by 2.89% and 11.39%, respectively, whereas MK50 exhibited a 69.16% reduction. During the acceleration period, the normalized heat flow values for the acceleration peaks of the reference group, MK10, and MK20 were 2.21 mW/g, 2.03 mW/g, and 1.62 mW/g, respectively, while MK50 did not exhibit a distinct acceleration peak. The peak intensities of MK10 and MK20 were reduced by 8.14% and 26.70%, respectively, compared with the reference group. In summary, the incorporation of MK delays the onset of the initial reaction period, extends the duration of the induction period, and slows the reaction rate during the acceleration phase. However, unlike the trends observed with FA and SF, moderate amounts of MK accelerate the reaction rate during the initial reaction period.

Figure 8b illustrates the influence of MK dosage on the cumulative hydration heat. As shown in the figure, after 24 h of cumulative heat release, the reference group exhibited a hydration heat of 114.4 J/g, while MK10, MK20, and MK50 showed hydration heats of 116.4 J/g, 111.0 J/g, and 55.2 J/g, respectively. MK10’s hydration heat increased by 1.75% compared with the reference group, whereas the MK20 and MK50 hydration heats decreased by 2.97% and 51.75%, respectively. After 72 h of cumulative heat release, the reference group reached a hydration heat of 144.8 J/g, while MK10, MK20, and MK50 achieved hydration heats of 148.5 J/g, 144.4 J/g, and 87.6 J/g, respectively. MK10’s hydration heat increased by 2.56% compared with the reference group, whereas the MK20 and MK50 hydration heats decreased by 0.28% and 39.50%, respectively.

### 3.4. Strength

#### 3.4.1. Compressive Strength

**Figure 9:**
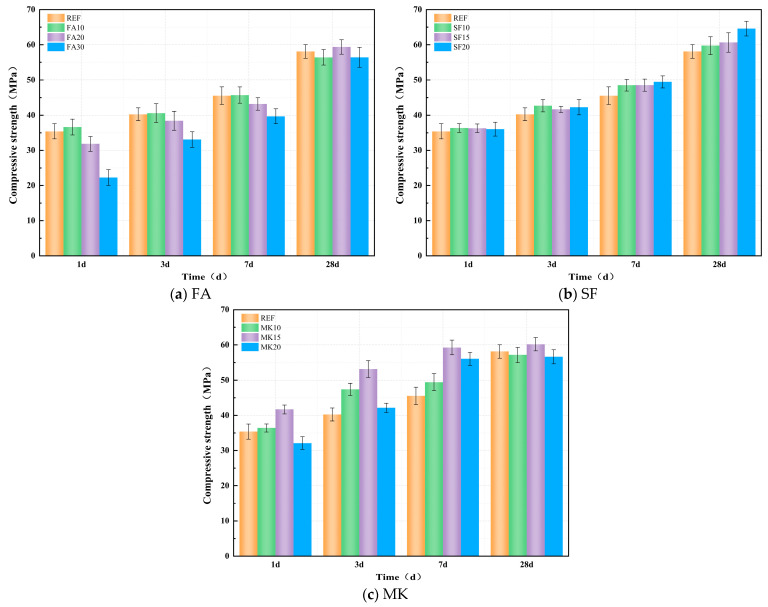
Effect of mineral admixture on compressive strength.

Figure 9a–c show the effect of FA, SF, and MK on the compressive strength of the paste. As shown in Figure 9a, the compressive strength generally increases with the FA dosage up to a certain point, after which it decreases, with the 10% dosage yielding the optimal performance. At a 10% FA dosage, the compressive strengths of the specimens at 1, 3, and 7 days increase by 3.42%, 0.85%, and 0.36%, respectively, compared with the reference group. However, increasing the FA dosage beyond 10% significantly impairs early-age compressive strength, with the negative impact becoming more pronounced at higher dosages. Specifically, FA20 and FA30 show 10.04% and 37.09% reductions in 1-day compressive strength, respectively, compared with the reference group. As the curing age progresses, the compressive strength growth rates of FA20 and FA30 specimens gradually accelerate. At 28 days, the compressive strength of FA30 is only 2.85% lower than that of the reference group (without FA), while FA20 achieves a 2.21% increase over the reference group. These results indicate that, at an appropriate FA dosage, the micro-aggregate effect and filling effect of FA improve the compactness of the paste, offsetting the adverse effects caused by its lower reactivity. With prolonged curing, the pozzolanic activity of FA is gradually activated under the continuous stimulation of alkali activators, enhancing the compressive strength growth rate. Consequently, the 28-day compressive strengths of specimens with different FA dosages become comparable to that of the reference group.

In Figure 9b, it can be observed that the compressive strengths of specimens with varying SF dosages consistently exceeded that of the reference group without SF. At 1 day, the compressive strengths of SF10, SF15, and SF20 were 2.71%, 2.49%, and 1.78% higher, respectively, compared with the reference group. As curing progressed, the compressive strength growth rates of the SF-modified specimens remained higher than those of the reference group, with the growth rate increasing proportionally to the SF dosage. After 28 days of curing, the compressive strengths of SF10, SF15, and SF20 were 2.86%, 4.39%, and 11.19% higher, respectively, than the reference group. Notably, SF20 exhibited the most pronounced enhancement. This improvement is attributed to SF’s high specific surface area and strong pozzolanic reactivity, which enable rapid reactions with alkali activators to generate substantial C-S-H gel, thereby enhancing the compactness of the hardened matrix. Simultaneously, higher SF dosages fully exploit the micro-filling effect, further refining the pore structure within the microstructure and contributing to the increased compressive strength of the matrix.

In Figure 9c, it can be observed that with the increase in MK dosage, the compressive strength initially increases and then decreases. Comparatively, the optimal effect is achieved at a 15% dosage. When the MK dosage is 15%, the compressive strength of specimens at various curing ages is higher than that of the reference group without MK. The 1 d, 3 d, 7 d, and 28 d compressive strengths are increased by 17.77%, 32.02%, 30.10%, and 3.55%, respectively, compared with the reference group. Meanwhile, as shown in the figure, MK exhibits a more pronounced enhancement effect on the early-age (≤7 d) compressive strength than on later stages at different curing ages, demonstrating a clear early strengthening effect. This indicates that at an appropriate MK dosage, under the sustained activation of alkaline activators, the highly reactive Al_2_O_3_ and SiO_2_ in MK are fully utilized to generate more gel products such as N-A-S-H. The spatial interconnection between N-A-S-H and C-A-S-H improves the compactness of the matrix, facilitating substantial growth in compressive strength. However, when the dosage exceeds 15%, the high water absorption of MK may lead to reduced slurry fluidity and increased internal defects, thereby decreasing compressive strength.

In summary, the incorporation of SF is more beneficial to the later-stage strength development of specimens, while MK demonstrates superior enhancement in early-age compressive strength. Although the addition of FA reduces early strength, it significantly accelerates the later strength growth rate, and the 28-day compressive strength does not exhibit significant loss.

#### 3.4.2. Splitting Tensile Strength

**Figure 10:**
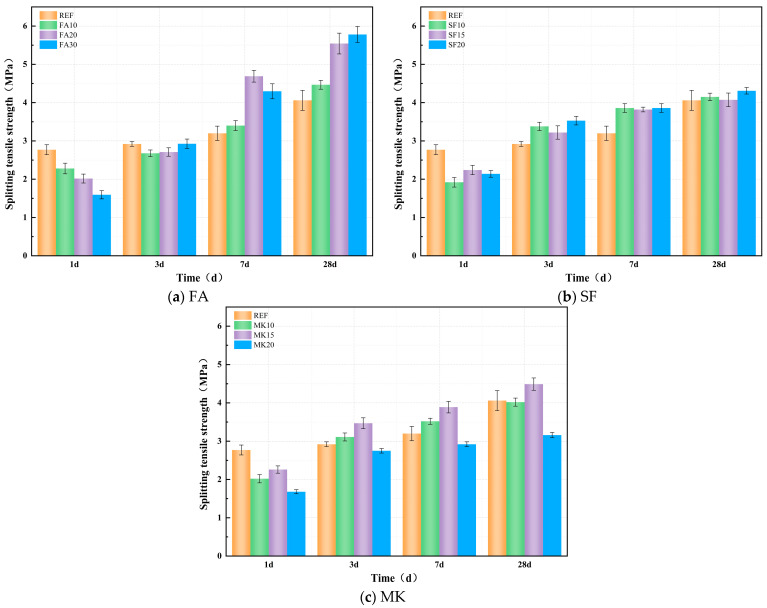
Effect of mineral admixture on splitting tensile strength.

Figure 10a–c show the effect of FA, SF, and MK on the splitting tensile strength of geopolymer paste. From Figure 10a, it can be observed that the incorporation of FA exhibits a two-stage development pattern in splitting tensile strength. During the early reaction stage, the splitting tensile strength of specimens decreases with increasing FA dosage. After 1 day, the splitting tensile strengths of FA10, FA20, and FA30 are 17.69%, 27.20%, and 42.43% lower than the reference group, respectively. As the curing age progresses, the growth rate of splitting tensile strength in FA-incorporated specimens gradually accelerates, and the strength increases with higher FA dosages. At 7 days, the splitting tensile strengths of FA10, FA20, and FA30 are 6.26%, 46.56%, and 34.23% higher than the reference group, respectively. At 28 days, the strengths of FA10, FA20, and FA30 exceed the reference group by 10.03%, 36.53%, and 42.40%, respectively. This phenomenon is attributed to the low pozzolanic activity of FA, which results in slower early-stage reactions. As curing progresses, the pozzolanic activity of FA gradually develops, facilitated by the sustained activation of alkaline activators. Additionally, the micro-aggregate filling effect of FA contributes to an increased packing density of the slurry, which improves the overall compactness of the matrix. This enhanced microstructure results in a continuous improvement in the splitting tensile strength of the material.

As observed in Figure 10b, the incorporation of SF results in a two-stage development pattern for splitting tensile strength. During the early reaction stage, the addition of SF reduces the splitting tensile strength of the specimens. After 1 day, the splitting tensile strengths of SF10, SF15, and SF20 are 30.69%, 19.13%, and 22.74% lower than the reference group, respectively. However, as curing progresses, the rate of increase in splitting tensile strength for SF-incorporated specimens gradually accelerates. At 7 days, the splitting tensile strengths of SF10, SF15, and SF20 are 20.63%, 19.38%, and 20.63% higher than the reference group, respectively. By 28 days, the strengths of SF10, SF15, and SF20 surpass the reference group by 2.22%, 0.25%, and 6.16%, respectively.

In Figure 10c, it can be observed that the incorporation of MK exhibits a similar trend in splitting tensile strength as that observed in compressive strength, generally showing an initial increase followed by a decrease. During the early reaction stage, the addition of MK reduces the splitting tensile strength of the specimens. After 1 day, the splitting tensile strengths of MK10, MK15, and MK20 are 27.08%, 18.41%, and 39.35% lower than the reference group, respectively. As the curing age progresses, the growth rate of splitting tensile strength in MK-incorporated specimens gradually accelerates. Notably, the 15% MK dosage yields the optimal effect. At 3 days, 7 days, and 28 days, the compressive strengths of the MK15 specimen exceeded those of the reference group by 18.84%, 21.56%, and 10.59%, respectively.

#### 3.4.3. Tension–Compression Ratio

**Figure 11:**
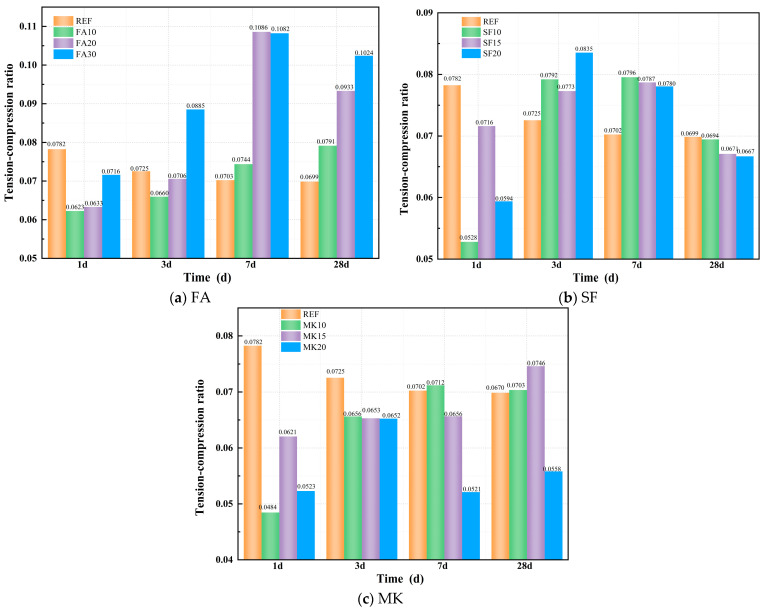
Effect of mineral admixture on tension–compression ratio.

Figure 11a–c show the effect of FA, SF, and MK on the tension–compression ratio of geopolymer paste. From Figure 11a, it can be observed that the incorporation of FA specimens shows that the tension–compression ratio is lower than that of the FA-free reference group during the early reaction stage (1 d). After 1 day, the tension–compression ratios of FA10, FA20, and FA30 decreased by 20.41%, 19.08%, and 8.47%, respectively, compared with the reference group. As the curing age progresses, both the compressive strength and splitting tensile strength growth rates of the FA-incorporated specimens gradually accelerate. In contrast, the later-stage increase in splitting tensile strength becomes more pronounced with higher FA dosages. By 28 days, the tension–compression ratios of FA10, FA20, and FA30 exceed the reference group by 13.28%, 33.58%, and 46.58%, respectively.

From Figure 11b, it can be observed that the incorporation of SF specimens exhibits a three-stage development pattern in the tension–compression ratio. During the early reaction stage, the tension–compression ratios are lower than those of the SF-free reference group. After 1 day, the ratios of SF10, SF15, and SF20 decreased by 32.52%, 21.10%, and 24.09%, respectively, compared with the reference group. As curing progresses, both compressive strength and splitting tensile strength growth rates in the SF-incorporated specimens accelerate. SF enhances the splitting tensile strength more than the compressive strength during the first 7 days. At 7 days, the tensile-to-compressive ratios of SF10, SF15, and SF20 exceed the reference group by 13.24%, 12.02%, and 11.11%, respectively.

From 7 to 28 days, SF enhances compressive strength more, resulting in reductions of 0.62%, 3.97%, and 4.52% in the tension–compression ratios of SF10, SF15, and SF20, respectively, compared with the reference group after 28 days of curing. From Figure 11c, it can be observed that the incorporation of MK specimens shows that the tension–compression ratios are lower than those of the MK-free reference group during the early reaction stage. After 1 day, the ratios of MK10, MK15, and MK20 decreased by 38.08%, 20.70%, and 33.16%, respectively, compared with the reference group. As the curing age progresses, MK exerts a more pronounced enhancement effect on splitting tensile strength. Consequently, after the standard-cured 28 days, the tension–compression ratios of MK10 and MK15 exceed the reference group by 0.70% and 6.80%, respectively, while that of MK20 is reduced by 20.12% compared with the reference group.

### 3.5. Five-Dimensional Evaluation

**Figure 12:**
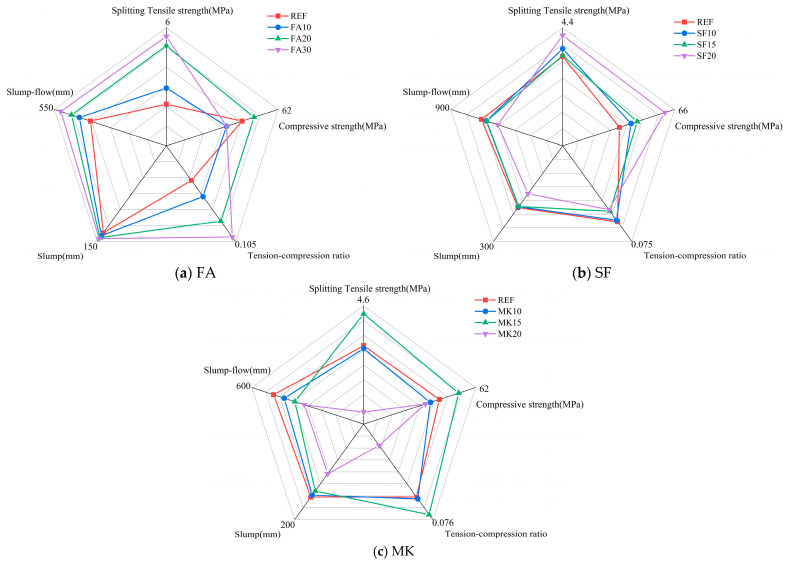
Five-dimensional evaluation diagram under different mineral admixtures.

Based on the experimental results, this study selected five indicators—compressive strength, splitting tensile strength, tension–compression ratio, slump, and slump flow spread—and conducted comparative analyses using a five-dimensional evaluation method [49]. The compressive strength and splitting tensile strength of the specimens were tested after 28 days of standard curing (20 ± 2 °C, ≥95% relative humidity). Through multi-dimensional assessment, the optimal dosages of FA, SF, and MK were proposed. The multi-dimensional evaluation of slag–Yellow River silt-based geopolymers with varying dosages of mineral admixtures is shown in Figure 12. As seen, a 30% FA dosage results in a significant improvement in workability, splitting tensile strength, and the tension–compression ratio compared with the reference group. The compressive strength is only 2.85% lower than the reference, indicating balanced overall performance. At a 20% SF dosage, the geopolymer demonstrates substantial improvements in both splitting tensile and compressive strengths, indicating superior mechanical properties. However, practical construction requires attention to the workability of the mixture to avoid negative impacts on forming quality. At a 15% MK dosage, the geopolymer shows notable improvements in splitting tensile strength, compressive strength, and tension–compression ratio, providing well-rounded mechanical performance.

## 4. Analysis of Microstructural Mechanisms

### 4.1. Characteristic Hydration Products

**Figure 13:**
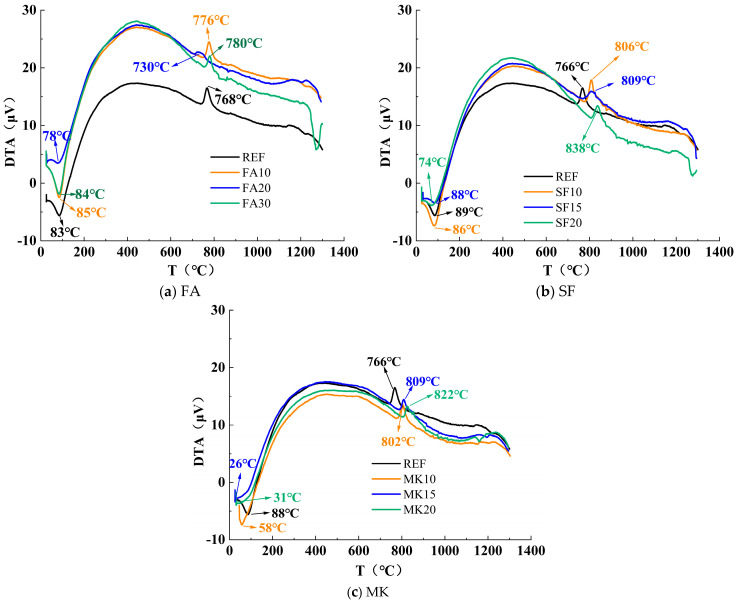
Comparative study of thermal analysis curves under different FA, SF, and MK contents.

Figure 13 presents the results of comprehensive thermal analysis under varying dosages of FA, SF, and MK. As shown in the figure, the thermal analysis curves exhibit two main thermal decomposition peaks, occurring within the temperature ranges of 60 °C–100 °C and 700 °C–850 °C [50,51]. The endothermic peak and mass loss in the 60 °C–100 °C range are primarily caused by the removal of free water from pore structures in the matrix and the release of interlayer and adsorbed water from gel-like hydration products. The exothermic peak and mass loss in the 700 °C–850 °C range are mainly attributed to the breaking of hydroxyl bonds within the structural framework of the gel products.

Figure 13a demonstrates that as the FA dosage increases, the mass loss rate around 80 °C rises significantly. This can be attributed to the water absorption capacity of FA, which increases both the free and adsorbed water content in the material. Additionally, the reaction between FA and alkaline activators leads to the formation of N-A-S-H gel, which adsorbs substantial amounts of free water, further exacerbating the mass loss.

At a 30% FA dosage, a new thermal decomposition peak emerges around 1370 °C, primarily due to the decomposition of the mullite (Al_6_Si_2_O_13_) present in FA at elevated temperatures, resulting in the formation of alumina (Al_2_O_3_) and silica (SiO_2_).

Figure 13b shows that as the SF dosage increases, the mass loss rate around 80 °C significantly rises. This is attributed to the extremely high specific surface area of SF (15,000–30,000 m^2^/kg), which enables it to adsorb more free water. Additionally, the high-temperature decomposition temperatures of the reference group, SF10, SF20, and SF30 are 766 °C, 806 °C, 809 °C, and 838 °C, respectively, demonstrating an increase in decomposition temperature with higher SF dosages. This phenomenon occurs because the highly reactive SiO_2_ in SF rapidly reacts with alkaline activators, generating more C-A-S-H gel and C-S-H gel with low calcium-to-silica ratios. These gels exhibit higher thermodynamic stability, thus significantly elevating the thermal decomposition temperature.

Figure 13c shows that, unlike FA and SF, the mass loss rate around 80 °C decreases with increasing MK dosage, and the endothermic peak shifts earlier compared with the reference group. This is attributed to the high reactivity of MK, which accelerates hydration reactions, converting more free and adsorbed water into bound water (gel water), thereby reducing the content of unbound water. Additionally, the high-temperature decomposition temperatures for the reference group, MK10, MK15, and MK20 are 766 °C, 801 °C, 809 °C, and 822 °C, respectively, indicating a progressive increase in decomposition temperature with higher MK dosages. This trend is attributed to the highly reactive Al_2_O_3_ and SiO_2_ in MK, which react with alkaline activators to form substantial amounts of N-A-S-H and C-A-S-H gels. These gels create tightly interconnected networks with improved thermodynamic stability, resulting in a significant increase in thermal decomposition temperatures.

**Figure 14:**
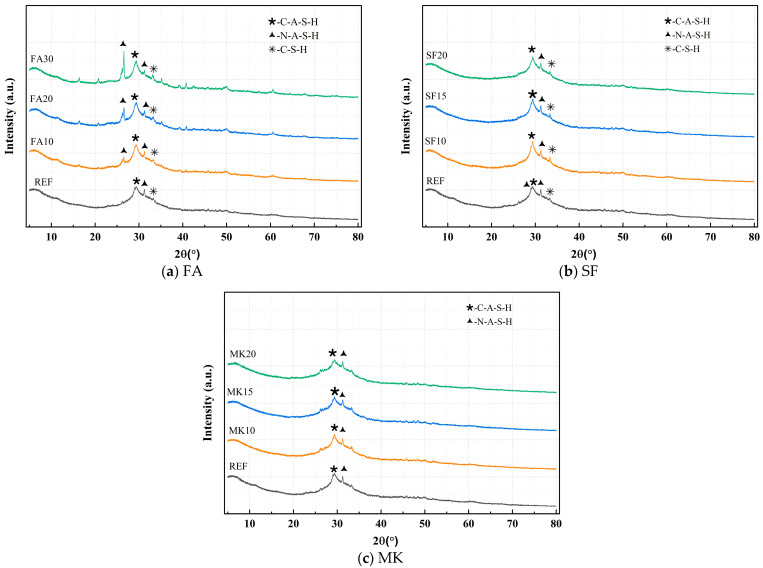
Comparative study of XRD patterns under different FA, SF, and MK contents.

Figure 14 shows the XRD curves under varying dosages of FA, SF, and MK. As seen in Figure 14a, with increasing FA dosage, the characteristic peak intensities of N-A-S-H strengthen, while those of the C-S-H and C-A-S-H gels weaken. This is because FA, classified as a low-calcium mineral admixture, contains only 7.23% CaO, significantly lower than the 41.06% CaO content in slag. The incorporation of FA dilutes the CaO content in the original system, resulting in reduced formation of C-S-H and C-A-S-H gels and a corresponding reduction in their diffraction peak intensities.

Figure 14b shows that as the SF dosage increases, the characteristic peak intensity of C-S-H gel significantly strengthens, while the intensity of the quartz (SiO_2_) peak weakens. This is attributed to the highly reactive SiO_2_ in SF, which rapidly reacts with alkaline activators to form substantial amounts of C-S-H gel, thereby enhancing its diffraction peak intensity. Simultaneously, the quartz in SF is consumed during the reaction process, resulting in a decrease in its diffraction peak intensity.

Figure 14c shows that with increasing MK dosage, the characteristic peak intensities of the N-A-S-H gel and calcium aluminate hydrate C-A-H strengthen, while those of the C-A-S-H gel weaken, and the diffraction peaks of C-S-H gel nearly disappear. Meanwhile, the characteristic peak intensities of quartz SiO_2_ and metakaolinite Al_2_Si_2_O₅(OH)₄ also diminish. This occurs because the Al_2_O_3_ and SiO_2_ in metakaolin react with alkaline activators to primarily generate N-A-S-H gel and C-A-H, leading to enhanced diffraction peaks for these phases. Concurrently, the quartz and metakaolinite in MK are consumed during the reaction, resulting in reduced diffraction peak intensities. Furthermore, the high aluminum content of MK creates a relative deficiency of CaO in the system, which suppresses the formation of C-S-H gel, causing its diffraction peaks to weaken or disappear entirely.

### 4.2. Matrix Microstructure

#### 4.2.1. Effect of FA Content on Matrix Microstructure

**Figure 15:**
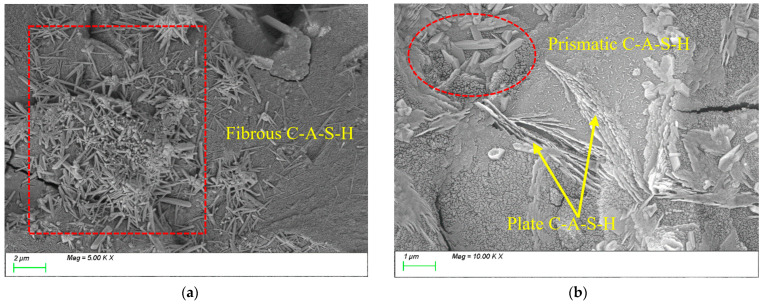
Microscopic images of REF.

Figure 15 presents the SEM results for the reference group. As shown, after 28 days of curing, numerous characteristic products with distinct morphologies have formed, exhibiting primarily three distribution patterns. Figure 15a shows the formation of abundant fibrous gels that interconnect to form a three-dimensional network structure. Figure 15b displays columnar and lamellar gels that are densely stacked, filling internal voids and refining the matrix porosity, thereby resulting in a compact structural framework.

**Figure 16:**
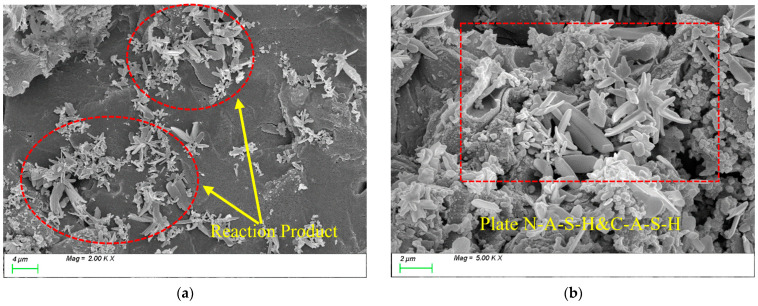
Microscopic images of FA-10.

Figure 16 shows the SEM testing results for the specimen with 10% FA incorporation. As seen in Figure 16a, the internal matrix exhibits sparse and underdeveloped product formation. Figure 16b reveals that the products are dominated by plate-like and flake-like gel phases. Energy spectrum analysis confirms the presence of N-A-S-H gel formed through the reaction between the aluminosilicate components in FA and alkaline activators. However, due to the relatively low reactivity of FA, the quantity of gel formed is limited, which could negatively affect the development of matrix strength.

**Figure 17:**
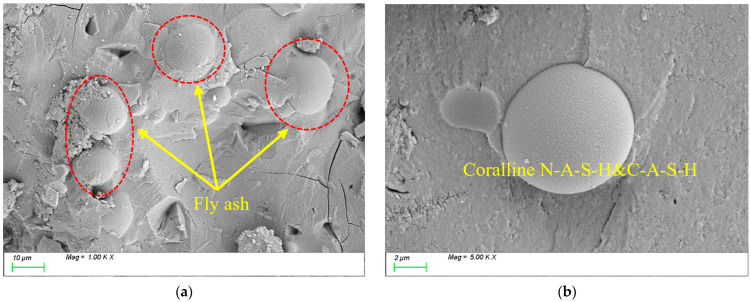
Microscopic images of FA-30.

Figure 17 presents the SEM results for the specimen with 30% FA incorporation. As shown in Figure 17a, partially unreacted FA particles remain visible, though the matrix structure appears relatively dense. Additionally, coral-like C-A-S-H gels, formed through pozzolanic reactions, are densely attached to the surfaces of spherical FA particles, as shown in Figure 17b. This suggests that alkaline activators effectively promote the pozzolanic reactions between slag and FA, resulting in the formation of abundant, dense hydration products such as C-A-S-H. These observations align with the conclusions drawn from the strength analyses.

#### 4.2.2. Effect of SF Content on Matrix Microstructure

**Figure 18:**
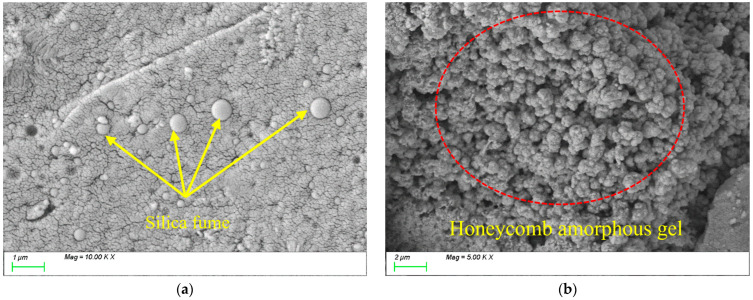
Microscopic images of SF-10.

Figure 18 presents the SEM testing results for SF10. As shown in Figure 18a, SF particles are predominantly spherical or near-spherical in shape with smooth surfaces. Figure 18b reveals that the C-S-H and C-A-S-H gels within the matrix still exhibit honeycomb-like growth characterized by a low degree of polymerization, resulting in a relatively loose structural framework.

**Figure 19:**
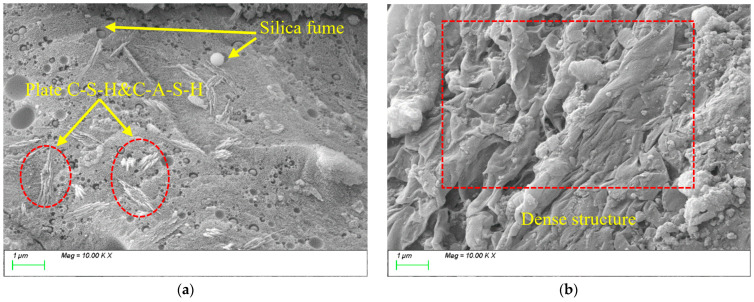
Microscopic images of SF-20.

Figure 19 presents the SEM results for SF20. As shown in Figure 19a, spherical SF particles remain visible, but their number and size in the matrix are significantly smaller compared with those in Figure 18a, indicating substantial participation of SF in the reaction. This is attributed to the extremely fine particle size and high specific surface area of SF. Under the activation of alkaline activators, these surface characteristics enhance the reactivity of SF, promoting its reaction with alkaline activators to form plate-like C-S-H and C-A-S-H gels. Figure 19b shows that the C-S-H gel particles in the matrix are tightly bound, forming a dense, blocky structure. This microstructural refinement is a key factor contributing to its significantly higher strength compared with the reference group.

#### 4.2.3. Effect of MK Content on Matrix Microstructure

**Figure 20:**
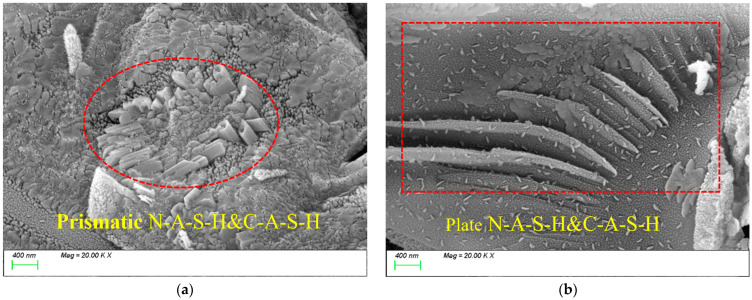
Microscopic images of MK-15.

Figure 20 presents the SEM testing results for MK15. As shown in Figure 20a, with the incorporation of MK, the characteristic products within the matrix are predominantly rod-like and plate-like N-A-S-H and C-A-S-H gels, with a reduced proportion of C-S-H gel. Figure 20b demonstrates that the C-A-S-H gel coexists with N-A-S-H gel, showing well-bonded particles and a dense internal matrix structure.

**Figure 21:**
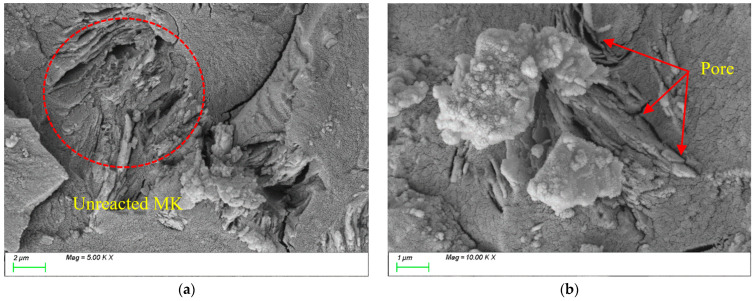
Microscopic images of MK-20.

Figure 21 presents the SEM testing results for MK20. As shown in Figure 21a, with the incorporation of a high MK dosage, numerous unreacted flaky MK particles are observed in the slurry. Due to the high specific surface area of the stacked flaky particles and insufficient filling of reaction products around them, the matrix develops a loose internal structure with increased porosity, which negatively impacts strength development, as shown in Figure 21b.

### 4.3. Pore Structure

**Figure 22:**
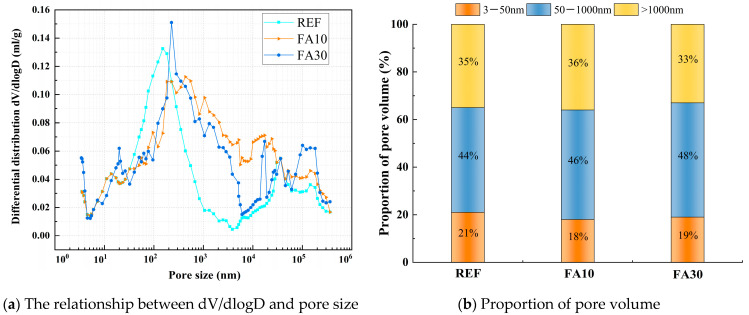
Comparative study of matrix pore characteristics under different FA contents.

**Figure 23:**
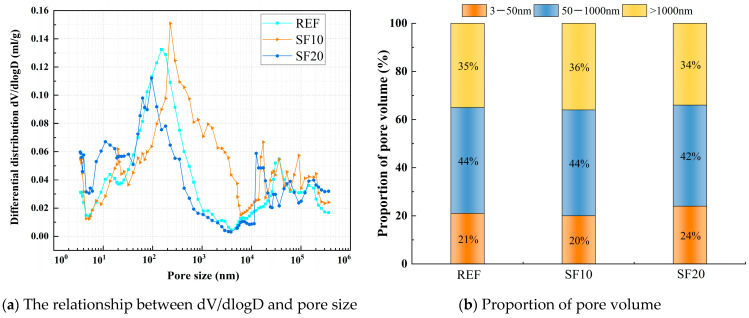
Comparative study of pore characteristics of the matrix under different SF contents.

**Figure 24:**
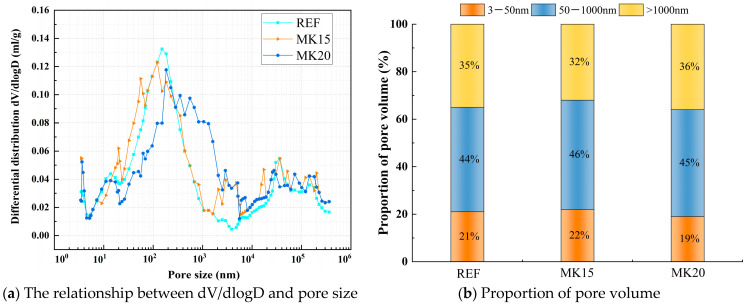
Comparative study of pore characteristics of matrix under different MK contents.

The effect of FA, SF, and MK contents on pore structures at an age of 28 d was selected for MIP experiments, as shown in Figure 22, Figure 23 and Figure 24. According to the results of the mercury injection test, the pore size is divided into three intervals: 3 nm–50 nm, 50–1000 nm, and >1000 nm. The pore structure exerts a significant influence on the matrix performance, and the mechanism of different sizes of pores has obvious differences. Gel pores (<50 nm) have a positive effect on the densification of the matrix. In the later curing process, gel pores not only can provide a channel for water migration, promote the continuous hydration of incompletely reacted mineral admixtures (such as slag, FA, SF, MK) to produce C-(A)-S-H gel and other products to refine the pore structure and improve the compactness of the matrix [52], which is conducive to the stable densification development of matrix microstructure, but also can significantly enhance long-term performance [53]. In contrast, macropores larger than 1000 nm are generally considered to be harmful pores, which will seriously affect the overall macroscopic properties of the material [54].

The relationship between dV/dlogD and the pore size of a matrix under different FA contents is shown in Figure 22a. The pore volume of a matrix under different FA contents is shown in Figure 22b. From Figure 22a, it can be observed that the most probable pore diameter of the reference group is 151 nm, while those of FA10 and FA30 are 227 nm and 433 nm, respectively. With increasing FA dosage, the most probable pore diameter initially increases and then decreases. Meanwhile, Figure 22b reveals that as the FA dosage increases, the proportions of gel pores and transition pores rise, while the proportion of macropores declines. When the FA dosage increases from 10% to 30%, the gel pore proportion grows from 18% to 19%, the transition pore proportion increases from 46% to 48%, and the macropore proportion decreases from 36% to 33%. Compared with the reference group, although FA30 exhibits a slightly lower gel pore proportion (resulting in marginally reduced compressive strength), its macropore fraction is significantly reduced, indicating a more homogeneous and denser material structure. This may be one of the main reasons why its splitting tensile strength surpasses that of the reference group.

The relationship between dV/dlogD and the pore size of a matrix under different SF contents is shown in Figure 23a. The pore volume of a matrix under different SF contents is shown in Figure 23b. From Figure 23a, it can be observed that the most probable pore diameter significantly decreases with increasing SF dosage. When the SF dosage reaches 20%, the most probable pore diameter drops to 95 nm, lower than the 151 nm of the reference group. Meanwhile, Figure 23b reveals that increasing the SF dosage notably enhances the proportion of gel pores. At a 20% SF dosage, the gel pore proportion increases from 21% to 24%, while the macropore proportion decreases from 35% to 34%. This suggests that incorporating an optimal amount of SF refines the pore structure of the matrix, contributing to enhanced material strength.

The relationship between dV/dlogD and the pore size of a matrix under different MK contents is shown in Figure 24a. The pore volume of the matrix under different MK contents is shown in Figure 24b. From the analysis of Figure 24a, it can be observed that the most probable pore diameters for MK15 and MK20 are 120 nm and 183 nm, respectively. As the MK dosage increases, the most probable pore diameter first decreases and then increases. Meanwhile, Figure 24b demonstrates that the proportions of gel pores and transition pores initially rise and then decline with higher MK dosages. For MK15, both gel pore and transition pore proportions exceed those of the reference group, while the macropore proportion is lower, which is one of the main reasons for its superior strength compared with the reference group. However, it is also noted that further increasing the MK dosage does not refine the pore structure of the matrix; instead, it has the opposite effect. When the MK dosage reaches 20%, the gel pore proportion decreases from 22% to 19%, and the macropore fraction increases from 32% to 36%. Therefore, selecting an optimal MK dosage is crucial for optimizing performance.

## 5. Conclusions

This study evaluated the impact of FA, SF, and MK on several critical properties of slag–YRS geopolymer, including the setting time, workability, compressive strength, splitting tensile strength, hydration heat, characteristic products, pore structure, and matrix microstructure. The following are the key findings of the study.

(1) In a strong alkali-activated environment, the setting times of all mixtures were relatively short, with minor differences. The incorporation of FA and MK delayed the setting process, while SF, due to its high specific surface area and reactivity, significantly accelerated the setting process.
(2) The workability of the mixtures was significantly influenced by the type and dosage of mineral admixtures. As the FA content increased, both slump and spread gradually improved. When the dosages of SF and MK increased from 20% to 30%, the slump dropped sharply from 136 mm and 125 mm to 75 mm and 80 mm, respectively. Therefore, it is recommended to control the dosages of SF and MK in practical applications to maintain optimal workability.
(3) The incorporation of FA, SF, and MK delayed the onset of the initial reaction period and extended the induction period. Additionally, FA and SF reduced the reaction rate during the initial reaction period, while MK slightly increased it. All three admixtures decreased the reaction rate during the acceleration period, thereby affecting the development of hydration heat, which is the main reason for the lower early compressive strength.
(4) The incorporation of mineral admixtures significantly enhanced the later strength of the matrix with varying effects. FA showed the greatest improvement in splitting tensile strength, with a 42.40% increase in the 28-day splitting tensile strength of FA30 compared with the control group. SF provided the best enhancement in compressive strength, with the 28-day compressive strength of SF20 increasing by 11.19% compared with the control group. The effect of MK was intermediate, with the 28-day compressive and splitting tensile strengths of MK15 increasing by 3.55% and 10.59%, respectively, compared with the control group.
(5) The optimal incorporation of FA, SF, and MK refined the pore structure and reduced the proportion of macropores in the matrix, though the mechanisms varied. FA promoted the formation of N-A-S-H gel, increasing the proportion of transitional pores (50–1000 nm). SF generated additional C-A-S-H gel and low-calcium C-S-H gel, while MK produced significant amounts of N-A-S-H and C-A-S-H gels, increasing the proportion of gel pores below 50 nm. These gels optimized the pore structure and enhanced matrix densification, contributing to microstructural reinforcement.
(6) Based on this study, FA can significantly improve the workability of geopolymers and provide more balanced heat release during hydration while maintaining satisfactory strength. Due to its high reactivity, SF effectively enhances matrix densification, making it the preferred supplementary material for high-strength engineering applications. MK not only improves strength but also has minimal adverse effects on workability, rendering it suitable for scenarios requiring a balance between strength and constructability. Although the experimental conditions of this study focused on investigating the effects of FA, SF, and MK on slag–Yellow River sediment-based geopolymers, the optimized parameter methodology is equally applicable to other aluminosilicate solid wastes (e.g., red mud, metallurgical slag, steel slag, and tailings). This research provides a novel solution for the disposal of aluminum industry waste and promotes the high-value utilization of solid waste resources.

## Figures and Tables

**Table 1:** Chemical compositions of YRS and mineral admixtures (wt.%).

Minerals	SiO_2_	CaO	Al_2_O_3_	Fe_2_O_3_	K_2_O	TiO_2_	MgO	Other
YRS	68.64	8.40	12.33	3.25	2.55	0.74	2.05	2.04
Slag	32.47	41.06	14.52	0.28	0.44	1.25	7.08	2.9
FA	45.88	7.23	20.15	16.55	4.21	2.43	0.20	3.35
SF	98.86	0.42	0.78	0.06	0.77	—	0.35	0.76
MK	53.57	—	44.40	0.94	0.73	0.19	0.09	0.08

**Table 2:** The chemical composition of sodium silicate.

SiO_2_/(%)	Na_2_O/(%)	H_2_O/(%)	Density/(g/cm^3^)	Modulus	Beaume
30	13.5	56.5	1.51	2.3	50

**Table 3:** Mix proportion for setting time and workability test.

No.	YRS	NaOH	SS	Slag	FA	MK	SF	Water
REF	1.000	0.020	0.128	0.660	—	—	—	0.192
FA10	1.000	0.020	0.128	0.594	0.066	—	—	0.192
FA20	1.000	0.020	0.128	0.528	0.132	—	—	0.192
FA30	1.000	0.020	0.128	0.462	0.198	—	—	0.192
MK10	1.000	0.020	0.128	0.594	—	0.066	—	0.192
MK20	1.000	0.020	0.128	0.528	—	0.132	—	0.192
MK30	1.000	0.020	0.128	0.462	—	0.198	—	0.192
SF10	1.000	0.020	0.128	0.594	—	—	0.066	0.192
SF20	1.000	0.020	0.128	0.528	—	—	0.132	0.192
SF30	1.000	0.020	0.128	0.462	—	—	0.198	0.192

**Table 4:** Mix proportion for hydration heat test.

No.	YRS	NaOH	SS	Slag	FA	MK	SF	Water
REF	1.000	0.020	0.128	0.660	—	—	—	0.192
FA10	1.000	0.020	0.128	0.594	0.066	—	—	0.192
FA20	1.000	0.020	0.128	0.528	0.132	—	—	0.192
FA30	1.000	0.020	0.128	0.462	0.198	—	—	0.192
FA50	1.000	0.020	0.128	0.330	0.330	—	—	0.192
MK10	1.000	0.020	0.128	0.594	—	0.066	—	0.192
MK20	1.000	0.020	0.128	0.528	—	0.132	—	0.192
MK50	1.000	0.020	0.128	0.330	—	0.330	—	0.192
SF10	1.000	0.020	0.128	0.594	—	—	0.066	0.192
SF20	1.000	0.020	0.128	0.528	—	—	0.132	0.192
SF50	1.000	0.020	0.128	0.330	—	—	0.330	0.192

**Table 5:** Mix proportion for strength and microcosmic test.

No.	YRS	NaOH	SS	Slag	FA	MK	SF	Water
REF	1.000	0.020	0.128	0.660	—	—	—	0.192
FA10	1.000	0.020	0.128	0.594	0.066	—	—	0.192
FA20	1.000	0.020	0.128	0.528	0.132	—	—	0.192
FA30	1.000	0.020	0.128	0.462	0.198	—	—	0.192
MK10	1.000	0.020	0.128	0.594	—	0.066	—	0.192
MK15	1.000	0.020	0.128	0.561	—	0.099	—	0.192
MK20	1.000	0.020	0.128	0.528	—	0.132	—	0.192
SF10	1.000	0.020	0.128	0.594	—	—	0.066	0.192
SF15	1.000	0.020	0.128	0.561	—	—	0.099	0.192
SF20	1.000	0.020	0.128	0.528	—	—	0.132	0.192

**Table 6:** Grouping of the workability, mechanical, reaction progress, and microstructural property tests.

Properties	Performance Index	Specimen Size	Quantity
Setting time	initial setting time	—	—
	final setting time	—	—
Workability	slump	—	—
	slump flow	—	—
Hydration heat	heat evolution rate	—	—
	accumulated hydration heat	—	—
Strength	compressive strength	100 mm × 100 mm × 100 mm	120
	splitting tensile strength	100 mm × 100 mm × 100 mm	120
Characteristic products	thermogravimetric analysis	40 mm × 40 mm × 40 mm	30
	X-ray diffraction analysis	40 mm × 40 mm × 40 mm	30
Microstructural properties	porosity	40 mm × 40 mm × 40 mm	30
	scanning electron microscopy	40 mm × 40 mm × 40 mm	30

## Data Availability

The original contributions presented in the study are included in the article; further inquiries can be directed to the corresponding authors.

## Nomenclature

FA	Fly ash	nm	Nanometer
YRS	Yellow River sediment	N	Newton
C-S-H	Calcium silicate hydrate	MgO	Magnesium Oxide
C-A-S-H	Calcium aluminum silicate hydrate	XRD	X-ray diffraction
SF	Silica fume	m	Meter
MIP	Mercury intrusion porosimetry	SiO_2_	Silicon dioxide
MK	Metakaolin	TiO_2_	Titanium dioxide
N-A-S-H	Sodium-Alumino-Silicate Hydrate	g·cm^−3^	Grams per cubic centimeter
EDS	Energy dispersive spectroscopy	${\mathrm{SO}}_{4}^{2-}$	Sulfate ion
OPC	Ordinary Portland cement		
TGA	Thermogravimetric analysis	SiO_2_	Silicon dioxide
SEM	Scanning electron microscope	SG	Specific gravity
AAFA	Alkali-activated fly ash	Min	Minute
EGC	Engineered geopolymer composites	MPa	Megapascal
mm	Millimeter	SS	Sodium silicate
m^2^	Square meters	NaOH	Sodium hydroxide
m^2^·kg^−1^	Square meters per kilogram	%	Percentage
